# Changes in Gray Matter Asymmetries of the Fusiform and Parahippocampal Gyruses in Patients With Subcortical Ischemic Vascular Disease

**DOI:** 10.3389/fneur.2020.603977

**Published:** 2021-01-21

**Authors:** Runtian Cheng, Li Chen, Xiaoshuang Liu, Tianyou Luo, Junwei Gong, Peiling Jiang

**Affiliations:** ^1^The Department of Radiology, The First Affiliated Hospital of Chongqing Medical University, Chongqing, China; ^2^The Department of Radiology, The Affiliated Hospital of North Sichuan Medical College, Nanchong, China; ^3^The Department of Radiology, The Second Affiliated Hospital of Military Medical University, Chongqing, China

**Keywords:** subcortical ischemic vascular disease, vascular cognitive impairment, gray matter asymmetry, MRI, voxel-based morphometry

## Abstract

**Objective:** Changes in the normal asymmetry of the human brain often mean pathology. Current studies on the correlation between asymmetry and cognitive impairment have focused on Alzheimer's disease (AD) and AD-related mild cognitive impairment (MCI). The purpose of this study was to investigate changes in gray matter asymmetry and their relationship with cognitive impairment in patients with subcortical ischemic vascular disease (SIVD) by using voxel-based morphological measurements.

**Methods:** Fifty-nine SIVD patients with (subcortical vascular cognitive impairment, SVCI, *N* = 30) and without (pre-SVCI, *N* = 29) cognitive impairment and 30 normal controls (NC, *N* = 30) underwent high-resolution structural MRI and neuropsychological examinations. The differences in gray matter asymmetry among the three groups were estimated by using one-way ANOVA. Moreover, partial correlation analysis was performed to explore the relationships between the asymmetry index (AI) values and cognitive assessments controlled for age, sex, and education.

**Results:** The gray matter asymmetries in the fusiform and parahippocampal gyruses of the SVCI group were significantly different from those of the NC group and the pre-SVCI group, while no differences were found between the NC group and the pre-SVCI group in the same areas. More specifically, in the fusiform and parahippocampal gyruses, the SVCI group displayed a dramatic rightward asymmetry, whereas the NC group and pre-SVCI group exhibited a marked leftward asymmetry. The results of the correlation analysis showed that the “mean AI” in significant cluster was strongly correlated with the changes in cognitive outcomes.

**Conclusion:** This study demonstrated different lateralization in the fusiform and parahippocampal gyruses of SIVD patients with cognitive impairment compared to healthy subjects and SIVD patients without cognitive decline. Our findings may contribute to better understanding the possible mechanism of cognitive impairment in patients with SIVD, and they suggest the possibility of using gray matter asymmetry as a biomarker for disease progression.

## Introduction

Vascular cognitive impairment (VCI) is a heterogeneous group of disorders caused by multiple vascular factors, including mild vascular cognitive impairment and vascular dementia (VaD) (1). Mild vascular cognitive impairment is considered to be the prodromal stage of VaD (2, 3), and VaD is the second most common type of dementia after Alzheimer's disease (AD) (4). Characterized by lacunar infarcts and profound white matter changes, subcortical ischemic vascular disease (SIVD), which is driven by small vessel disease, is the primary cause of VCI (5, 6). The prodromal stages of SIVD include subcortical ischemic vascular cognitive impairment without dementia and subcortical vascular mild cognitive impairment (7), in which individuals exhibit evidence of relevant vascular risk factors. In the early stage of SIVD, there may be no decline in cognitive function (2), and its mild cognitive impairment features include memory deficits that show a trend of gradual worsening. Therefore, early diagnosis and intervention in the prodromal stage are of great significance for clinical outcomes.

There are functional and structural asymmetries between the right and left hemispheres of the human brain (8, 9). Normal asymmetry is often the basis of functional coupling in the human brain (10). Changes in the normal asymmetry of the brain often mean pathological changes have taken place. For example, changes in the normal asymmetry of the brain have been found in patients with mental disorders (11). Numerous studies have previously reported various regional abnormalities of hemispheric asymmetry of the brain in AD and mild cognitive impairment (MCI), including changes in the cortical thickness (8), gray matter volume (12), white matter signaling (13), functional connectivity (14–18), and metabolism (19). It is generally accepted that a change of cortical asymmetry is closely related to alterations of human cognitive function (20, 21). However, previous studies on asymmetry of the brain have mostly focused on AD and AD-related MCI patients, and few studies have been conducted on patients with vascular cognitive impairment.

More recently, Kurth et al. (22) have provided a novel voxel-based morphological method for assessing gray matter asymmetry. Through spatial normalization into a symmetric space using the Diffeomorphic Anatomical Registration Through Exponentiated Lie (DARTEL) tool, this method can establish an accurate voxel-wise correspondence across individuals and across both hemispheres of the brain, distinct from the traditional direct left-right comparison, and obtain both the direction and magnitude of asymmetry. This method has been used in several studies (23–26). In this study, the method was used to evaluate whether there was a change in gray matter asymmetry in SIVD patients and to further explore its relationship with cognitive impairment.

## Materials and Methods

### Participants

In this study, 89 participants, including 59 SIVD patients with (subcortical vascular cognitive impairment, SVCI, *N* = 30) and without (pre-SVCI, *N* = 29) cognitive impairment and normal controls matched for age, sex and education (NC, *N* = 30), were recruited between 2018 and 2020 at the First Affiliated Hospital of Chongqing Medical University. Informed consent was obtained from each subject. Furthermore, the study was approved by the ethics committee of the institution.

The inclusion criteria for the patients with SIVD were as follows (27): (1) white matter hyperintensities extending into the deep white matter; periventricular abnormalities, extending caps (> 10 mm as measured parallel to the ventricle) or an irregular halo (> 10 mm with irregular margins and extending into the deep white matter); and diffusely confluent hyperintensities (> 25 mm with an irregular shape) or extensive white matter impairment; (2) multiple lacunar lesions (> 5) in the deep gray matter and at least moderate white matter lesions; and (3) lack of hemorrhages, cortical and/or territorial infarcts and watershed infarcts, signs of normal pressure hydrocephalus, and definite causes of the white matter lesions.

The criteria for the SVCI group were as follows (28): (1) subjective cognitive complaints reported by the participant or his/her caregiver; (2) cognitive impairment that does not meet the standard of the Diagnostic and Statistical Manual of Mental Disorders, fifth edition (DSM-V) criteria for dementia; (3) a Clinical Dementia Rating Scale (CDR) score = 0.5; and (4) a Mini-Mental State Examination (MMSE) score ≥ 24.

The pre-SVCI group met the following criteria: (1) absence of any impairment of daily life activities and cognitive assessments; (2) a CDR score = 0 and (3) a MMSE score ≥ 27.

The criteria for the NC included: (1) a lack of neurological and psychiatric disorders; (2) a lack of abnormal findings on conventional brain MR imaging and (3) no cognitive complaints.

Subjects were excluded if they showed one or more of the following: (1) abnormal metabolic conditions, such as hypothyroidism or folic acid deficiencies; (2) depression, schizophrenia, or any other psychiatric disorders; or (3) Parkinson's syndrome, epilepsy, or other nervous system diseases that influence cognitive function. Subjects with a relevant MR scanning contraindication or metallic foreign body were excluded from the study.

### Neuropsychological Assessment

All participants were administered a comprehensive neuropsychological assessment battery including the following aspects: Mini-Mental Status Examination (MMSE) for global cognition, Auditory Verbal Learning Test (AVLT) for episodic memory, Boston Naming Test (BNT) for language function, Clock Drawing Test (CDT) for visuospatial perception, Trail Making Tests A and B (TMT-A and TMT-B) for executive functions, Rey-Osterrieth Complex Figure Test (CFT) for visual memory, Stroop Color Word Test (Stroop1 and Stroop2) for working memory. Each participant's raw neuropsychological data were transformed into z-scores.

### MRI Acquisition

All the MRI data were acquired on a GE Signa Hdxt 3.0T scanner with an eight-channel phased-array head coil. High-resolution 3D-T1 images were obtained with the following parameters: TR = 8.3 ms, TE = 3.3 ms, flip angle = 15°, thickness/gap = 1.0/0 mm, field of view (FOV) = 240 × 240 mm, matrix = 240 × 240, voxel = 1 × 1 × 1 mm^3^, and scanning time = 6.45 min. The scan parameters of the T2-FLAIR-weighted images were acquired as follows: TR = 8000 ms, TE = 126 ms, TI = 1500 ms, thickness/gap = 5.0/1.5 mm, FOV = 240 × 240 mm, and matrix = 256 × 192. The subjects were told to keep their eyes closed and to remain awake throughout the scanning session.

### Data Preprocessing

To ensure the accuracy of the segmentation of the gray and white matter, lesions were first segmented by the lesion growth algorithm (29) as implemented in the LST toolbox version 1.2.3 (https://www.applied-statistics.de/lst.html) of the Statistical Parametric Mapping software (SPM, http://www.fil.ion.ucl.ac.uk/spm). Following the automated steps of the LST pipeline, the algorithm first segmented the T1 images into cerebrospinal fluid, gray matter and white matter. This information was combined with the coregistered FLAIR intensities to calculate lesion belief maps. Twenty values of the original initial threshold (κ, in the range of 0.05 to 1.0 with an interval of 0.05) were set, and an optimal κ value (κ = 0.35) was selected after visual inspection to ensure that the algorithm captured most lesions without segmenting nonlesion areas. By thresholding these maps with the selected optimal κ value, initial binary lesion maps were obtained that were subsequently grown along voxels that appeared hyperintense in the FLAIR images. Then, lesion probability maps were garnered and used to perform lesions filling in the high-resolution T1 images and lesions volume calculations.

The gray matter asymmetry analysis of the high-resolution 3D-T1 images after white matter lesion filling followed an established protocol for voxel-wise asymmetry analyses using the VBM8 toolbox (http://dbm.neuro.uni-jena.de/wordpress/vbm/) implemented in SPM8. Briefly, this procedure first segmented T1 images into separate gray and white matter and registered them into MNI space by applying 12 affine parameter transformations. Then, the affine registered gray and white matter segments were flipped at the midline in the sagittal plane, and a DARTEL template was created using the original and flipped affine registered segments. Next, the registered flipped and original gray matter segments were normalized to the symmetric DARTEL template. Subsequently, the asymmetry index (AI) on each voxel was calculated as AI = ([right–left]/[0.5 × [right+left]]). Ultimately, the left hemispheres were discarded, and a Gaussian kernel of 8 mm full-width-at-half-maximum was used to smooth the right hemispheres. The resulting smoothed right-hemispheric AI images established the input data for subsequent statistical analysis. The positive AI values of the remaining right hemisphere indicated a rightward asymmetry, while negative AI values indicated leftward asymmetry.

### Statistical Analyses

All the clinical and demographic data were evaluated among the groups using SPSS version 23. One-way ANOVA and *post hoc* multiple comparisons tests were used to assess the differences in continuous variables and cognitive assessment values after z-scores transformation across the three groups, and the χ^2^ test was used for the sex proportions.

The voxel-wise gray matter asymmetry differences among the three groups were examined via a general linear model with the covariates of interest, including age, sex, years of education, and volume of white matter hyperintensities (WMH). *Post hoc* tests were further used to detect specific differences between every two groups. All findings resulting from the group comparisons were corrected for multiple comparisons using nonparametric threshold-free cluster enhancement (TFCE) with 5,000 permutations and while controlling the family-wise error (FWE) rate at *p* < 0.05. Partial correlation analysis was performed to explore the relationships between the AI values extracted from the significantly different gray matter regions and thirteen cognitive assessments controlled for age, sex, and education (*P* < 0.05, two-tailed). Furthermore, the *P* values were adjusted by a Bonferroni correction due to multiple testing.

## Results

### Demographic and Cognitive Characteristics

The demographic and clinical characteristics of all the subjects are shown in Table 1. There were no significant differences in age, sex, or education among the different groups. The WMH volume was significantly different in the SVCI group and pre-SVCI group than in the NC group, but it was not substantially different between the SVCI group and the pre-SVCI group.

**Table 1:** Demographic characteristics of the participants in the three groups.

	**NC (*N* = 30)**	**pre-SVCI (*N* = 29)**	**SVCI (*N* = 30)**	***P***
Gender (M/F)	16(14)	19(10)	18(12)	0.112
Age (year)	67.36 ± 7.08	70.62 ± 3.81	70.13 ± 5.31	0.058
Education (year)	11.00 ± 2.70	11.34 ± 3.17	9.93 ± 1.76	0.100
WMH	−1.11 ± 0.10	0.45 ± 0.67^a^	0.68 ± 0.79^a^	<0.001^*^

*The differences of continuous variables across the groups were assessed by one-way ANOVA and sex proportions by the chi-square test. The data are presented as the mean ± SD for normally distributed variables*.

* *Significant by one-way ANOVA, P < 0.05. WMH, white matter hyperintensity*.

a *Significant compared with NC, P < 0.05*.

Table 2 displays the results of the cognitive assessments. The SVCI group exhibited the worst results on all the cognitive evaluation tests compared with the other two groups, while the pre-SVCI group was significantly worse on the TMT and the AVLT than the NC group.

**Table 2:** Cognitive characteristics of the participants in the three groups.

	**NC (*N* = 30)**	**pre-SVCI (*N* = 29)**	**SVCI (*N* = 30)**	***F/P* value**
MMSE	28.50 ± 1.11	28.10 ± 1.05	24.17 ± 1.90^a^^,^^b^	86.56/ <0.001^*^
AVLT-IR	0.75 ± 0.56	0.09 ± 0.79^a^	−0.84 ± 0.89^a^^,^^b^	33.32/ <0.001^*^
AVLT-DR	0.73 ± 0.59	0.13 ± 0.89^a^	−0.85 ± 0.78^a^^,^^b^	32.81/ <0.001^*^
AVLT-RR	0.74 ± 0.62	0.13 ± 0.89^a^	−0.87 ± 0.76^a^^,^^b^	35.84/ <0.001^*^
BNT	0.35 ± 0.71	0.35 ± 0.89	−0.69 ±1.02^a^^,^^b^	13.90/ <0.001^*^
CDT	0.27 ± 0.74	0.25 ± 1.06	−0.51 ± 1.00^a^^,^^b^	6.60/0.002^*^
TMT-A	−0.67 ± 0.50	−0.10 ± 0.95^a^	0.79 ± 0.90^a^^,^^b^	24.61/ <0.001^*^
TMT-B	−0.85 ± 0.44	0.05 ± 0.76^a^	0.83 ± 0.89^a^^,^^b^	40.59/ <0.001^*^
Rey CFT-IR	0.40 ± 0.43	0.11 ± 0.86	−0.53 ± 1.31^a^^,^^b^	7.35/0.001^*^
Rey CFT-DR	0.50 ± 0.93	0.21 ± 0.94	−0.72 ± 0.69^a^^,^^b^	15.602/ <0.001^*^
Stroop-1	0.50 ± 0.36	0.39 ± 0.55	−0.94 ± 1.17^a^^,^^b^	30.48/ <0.001^*^
Stroop-2	0.61 ± 0.67	0.17 ± 0.80	−0.83 ± 0.94^a^^,^^b^	23.11/ <0.001^*^

*The differences in continuous variables across the groups were assessed by one-way ANOVA. The data are presented as the mean ± SD for normally distributed variables*.

* *Significant by one-way ANOVA, P < 0.05*.

a *Significant compared with NC, P < 0.05 (Bonferroni corrected)*.

b *Significant compared with pre-SVCI, P < 0.05 (Bonferroni corrected). MMSE, Mini-Mental State Examination; AVLT-IR & AVLT-DR & AVLT-RR, Auditory Verbal Learning Test, immediate recall, delayed recall, and recognition recall; BNT, Boston Naming Test; CDT, Clock Drawing Test; TMT, Trail Making Test; Rey CFT-IR & DR, Rey complex figure test, immediate recall, and delayed recall. NC, normal controls; pre-SVCI, subcortical ischemic vascular disease without cognitive impairments; SVCI, subcortical ischemic vascular disease with cognitive impairments*.

### Group Differences in Gray Matter

**Figure 1:**
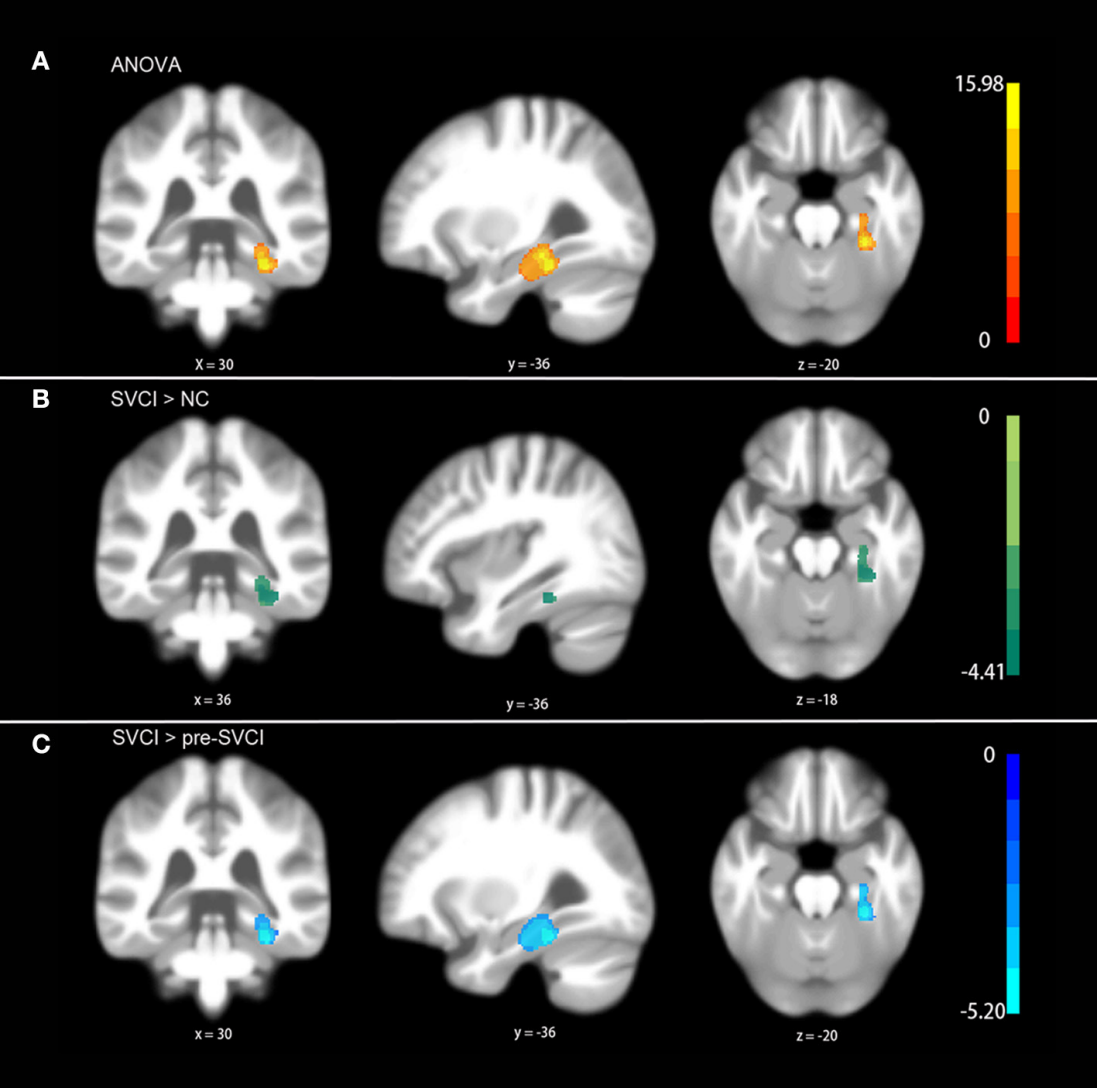
Differences in gray matter asymmetries among groups. **(A)** One-way ANOVA showed the differences in the fusiform and parahippocampal gyruses among groups. **(B)** The *post hoc* test indicated differences between the SVCI group and NC group. **(C)** The *post hoc* test suggested significant differences in the SVCI group compared with the pre-SVCI group. No differences were found between the NC group and the pre-SVCI group. The displayed template was generated from all of the subjects. All the results were corrected for multiple comparisons using a nonparametric threshold-free cluster enhancement with 5,000 permutations while controlling the family-wise error rate at *p* < 0.05.

As shown in Figure 1, we observed significant differences among the three groups regarding the gray matter asymmetry in the fusiform and parahippocampal gyruses (Figure 1A) by ANOVA controlled for age, sex, education, and volume of the WMH (*P* < 0.05, FWE corrected).

More specifically, the results of the *post hoc* test demonstrated that the gray matter asymmetries in the fusiform and parahippocampal gyruses of the SVCI group were significantly different from those of the NC group as well as the pre-SVCI group (Figures 1B,C) (*P* < 0.05, FWE corrected). Moreover, no differences were found between the NC group and the pre-SVCI group concerning the gray matter asymmetry in the areas that showed differences after ANOVA.

Subsequently, we extracted the voxel-wise AIs and the voxel-wise gray matter volumes from the cluster with significant differences to examine the causes of the detected group effect. Then, the averaged values over the entire significance cluster were calculated, and the cluster-specific “mean AI” and cluster-specific gray matter volumes for the left and right hemispheres were generated.

**Figure 2:**
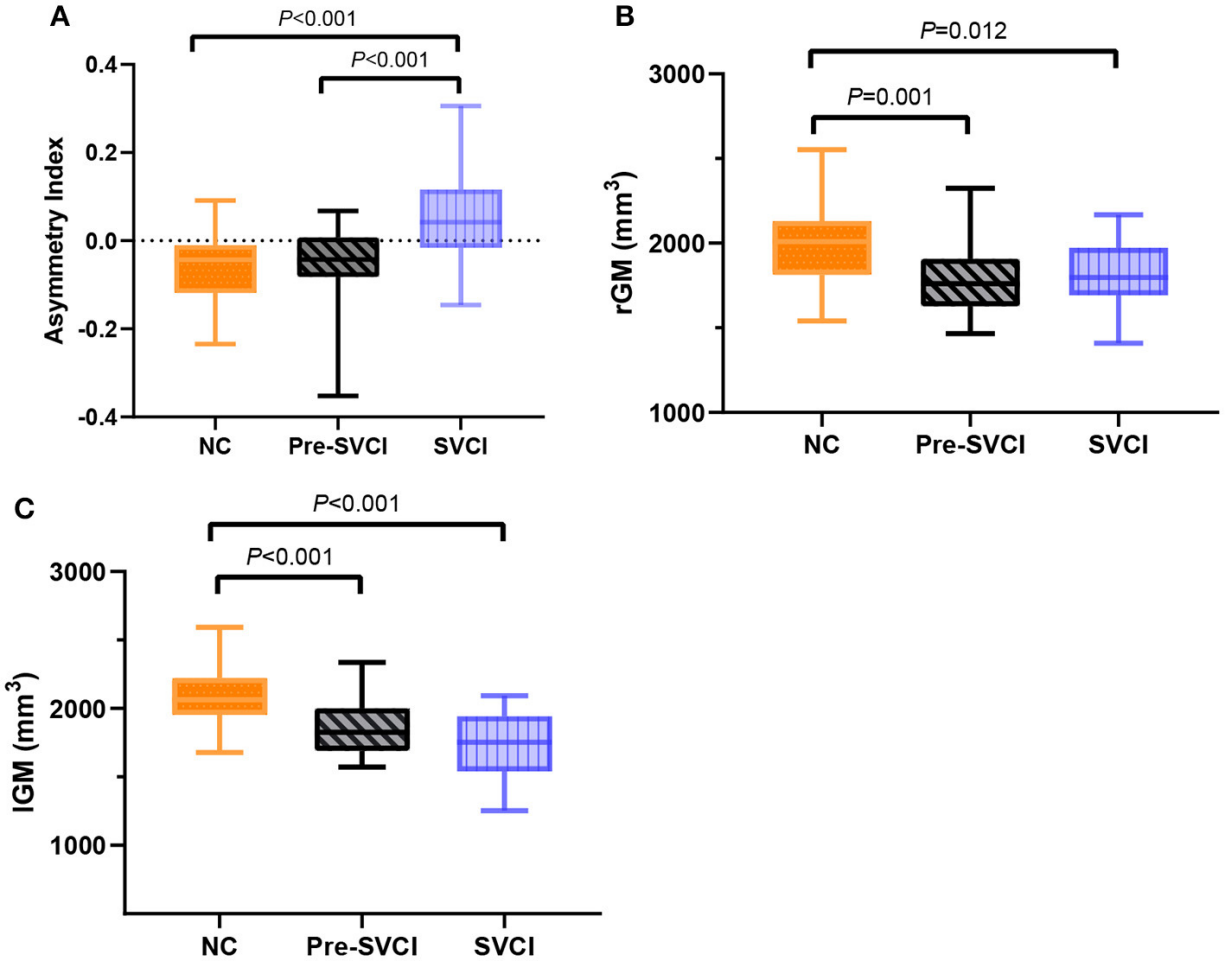
**(A)** The cluster-specific “mean AI” in the fusiform and parahippocampal gyruses among three groups. Positive values represent a rightward asymmetry; negative values represent a leftward asymmetry. **(B)** The cluster-specific “gray matter volumes” in the fusiform and parahippocampal gyruses in the right hemisphere. **(C)** The cluster-specific “gray matter volumes” in the fusiform and parahippocampal gyruses in the left hemisphere. The *P*-values indicate the significance of the group differences. AI, asymmetry index; rGM, The cluster-specific “gray matter volumes” in the right hemisphere; lGM, The cluster-specific “gray matter volumes” in the left hemisphere.

Taking the cluster-specific “mean AI” separately for each group, the SVCI group had a prominent rightward asymmetry, whereas the NC group and the pre-SVCI group showed a clear leftward asymmetry (Figure 2A). Moreover, when plotting the cluster-specific “gray matter volumes,” the pre-SVCI group and the SVCI group had significantly less gray matter than the NC group in both the right and left hemispheres, whereas these two groups had similar gray matter volumes in both regions (Figures 2B,C).

### Correlations With Cognitive Assessment

**Figure 3:**
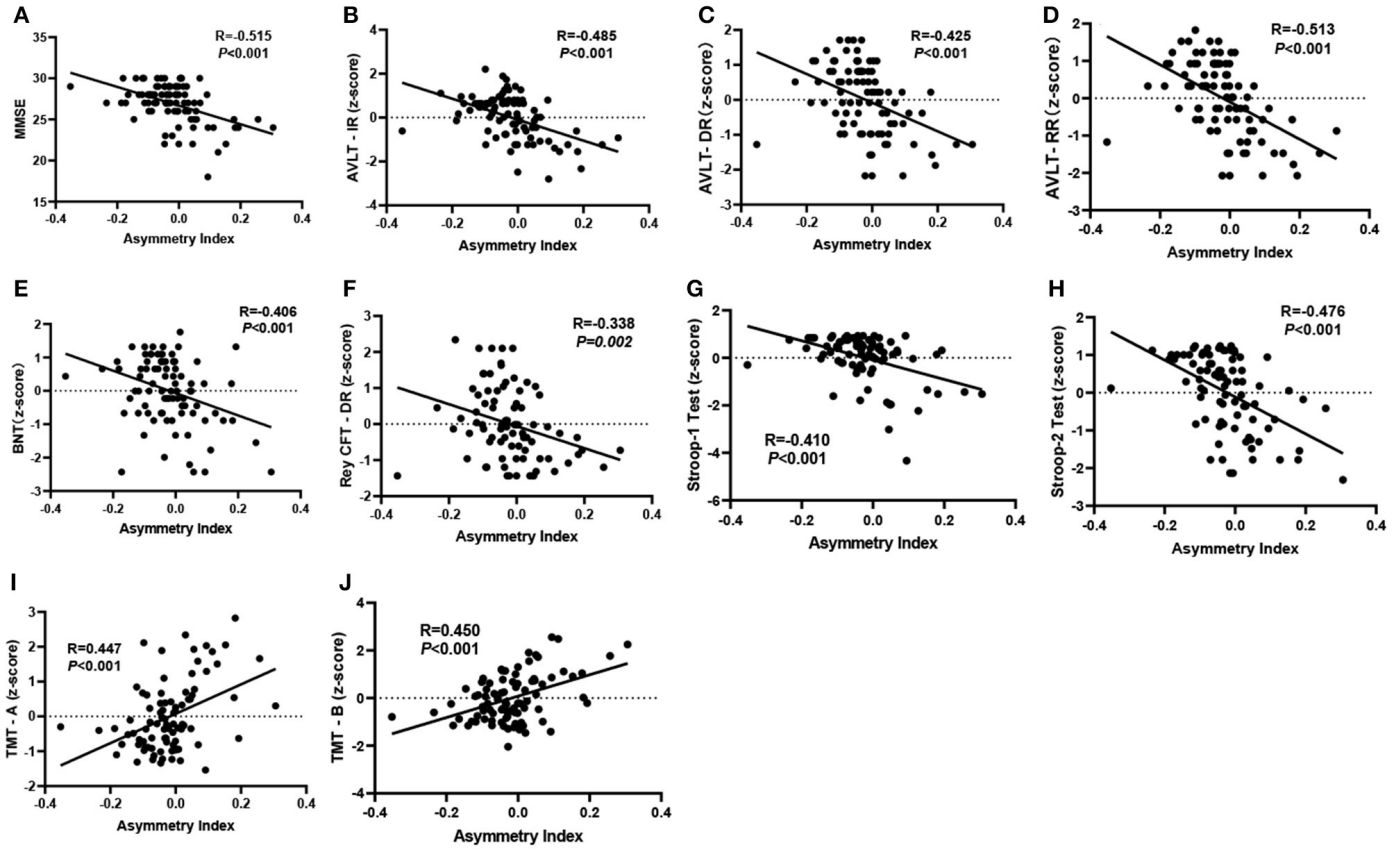
**(A–J)** Significant partial correlation between the “mean AI” in significant cluster and cognitive assessments controlled for age, sex, and education (*P* < 0.05, two-tailed). The *P* values were adjusted by Bonferroni correction. MMSE, Mini-Mental State Examination; AVLT-IR & AVLT-DR & AVLT-RR, Auditory Verbal Learning Test, immediate recall, delayed recall, and recognition recall; BNT, Boston Naming Test; TMT, Trail Making Test; Rey CFT DR, Rey complex figure test, delayed recall.

As shown in Figure 3, when relating the gray matter asymmetry to the cognitive assessment, an increase in the cluster-specific “mean AI” was firmly associated with decreases of the scores on the MMSE test (*r* = −0.515, *P* < 0.001), AVLT immediate recall (*r* = −0.485, *P* < 0.001), AVLT delayed recall (*r* = −0.425, *P* < 0.001), AVLT recognition recall (*r* = −0.513, *P* < 0.001), BNT (*r* = −0.406, *P* < 0.001), CFT delayed recall (*r* = −0.338, *P* = 0.002), Stroop1 test (*r* = −0.410, *P* < 0.001), and Stroop2 test (*r* = −0.476, *P* < 0.001). There was also a significant positive correlation between the cluster-specific “mean AI” and TMT, both for A (*r* = 0.447, *P* < 0.001) and B (*r* = 0.450, *P* < 0.001). All the *P* values were adjusted by Bonferroni correction.

## Discussion

This study was conducted to investigate the relationship between the changes in gray matter asymmetry and the decline of cognitive function in patients with SIVD. Our analysis revealed a prominent rightward gray matter asymmetry in the SVCI group compared with a leftward asymmetry in both the NC and pre-SVCI group within the specific areas of the fusiform and parahippocampal gyruses. We also observed that the gray matter asymmetry was significantly associated with cognitive decline, consisting of impairments in general cognitive function, episodic memory, and processing speed.

Asymmetry plays an essential role in the healthy human brain, and changes in standard asymmetry patterns often mean pathological changes in the brain. In recent years, increasing numbers of studies have focused on the relationship between abnormalities in brain symmetry and changes in the cognitive condition. These studies have explored the potential of using asymmetry as a biological marker in the development of cognitive disorders from all aspects of metabolism, function, and structure (30–32).

Previous studies have suggested that MCI patients with low metabolic status in the left hemisphere tend to exhibit more severe speech memory dysfunction with an increased risk of eventually being diagnosed with dementia (19). Liao et al. (31) found that asymmetry of functional brain activation might be a sensitive neuroimaging biomarker in the progression of MCI to dementia. Given the volume asymmetry of the bilateral hippocampus, the researchers found that the volume asymmetry in the hippocampus of patients with mild cognitive impairment and Alzheimer's disease was significantly different from that of healthy controls, showing different subpatterns of lateralization (10). Kim et al. (8) found that the cortex's asymmetry was significantly different between healthy people and patients with varying degrees of cognitive impairment. The asymmetry of the gray matter between the hemispheres in specific brain regions can provide more useful information than cortical volume and thickness measurements in predicting the conversion of MCI to AD (21).

In the past, most studies on the asymmetry of brain gray matter in patients with cognitive impairment have focused on AD and AD-related MCI, but little is known about the changes in asymmetry related to vascular cognitive impairment. Our study focused on the asymmetry of gray matter in the brains of SIVD patients. We found that the asymmetry in the gray matter volume of the fusiform and parahippocampal gyruses in the SVCI group accompanied by a decline in cognitive status was significantly different from that in the NC group and the pre-SVCI group. The SVCI group showed a rightward asymmetry in the fusiform and parahippocampal gyruses, while the NC and the pre-SVCI group showed a leftward asymmetry. Moreover, the asymmetry index was correlated with the results of multiple cognitive fields. These results suggested the possibility of using the gray matter asymmetry in the fusiform and parahippocampal gyruses as a new neuroimaging biomarker for tracking the progression of cognitive decline in patients with SIVD and for studying the underlying mechanism.

The fusiform gyrus has been shown to play an important role in facial recognition and in distinguishing facial expressions (33, 34). Previous studies have shown that patients with mild cognitive impairment have a reduced ability to handle facial information stimulation (35). The volume of the left fusiform gyrus in patients with mild cognitive impairment decreases with the development of the disease, accompanying the decline of cognitive function (36–38). This finding is consistent with the results of a previous study on cortical thickness (39). Our results showed that patients with SIVD had significant gray matter volume reductions in both the left and right fusiform gyruses compared with the NC. More specifically, SVCI patients showed a significant asymmetry to the right, while pre-SVCI patients showed a significant asymmetry to the left, which might indicate that the volume of the left area decreased more significantly in SVCI patients than in pre-SVCI patients.

The parahippocampal gyrus is generally considered to play an essential role in episodic memory, spatial analysis, and contextual association processing in many previous neuroimaging studies (40–44). Previous studies have shown that the gray matter volume of the hippocampus and the parahippocampus can effectively distinguish AD from healthy controls (45). Multiple studies have shown that subjects with MCI have less gray matter volume in the left parahippocampal gyrus than healthy elderly subjects (38). In this study, similar to the changes in the fusiform gyrus, we also found that the gray matter volume of the bilateral parahippocampal gyrus in patients with SIVD decreased substantially. These results are supported by outcomes reported by other researchers (46).

It is worth noting that although we observed a significant reduction of gray matter volume in the fusiform and parahippocampal gyruses in patients with SIVD compared to healthy controls, no differences were observed between the pre-SVCI and SVCI groups. The reason for this finding was most likely that the gray matter we were interested in was confined to regions with significant differences in asymmetry rather than a broader range.

Several limitations of this study deserve attention. First, the sample size of this study was small, which increased the statistical efficiency. Additional studies are needed to confirm the current results in a larger sample, which may also help to exclude differences in outcomes due to innate differences in the subjects. Second, although we found a significant correlation between gray matter asymmetry and cognitive decline, the underlying mechanisms and development trends of SIVD did not seem to be fully illustrated, given that this was a cross-sectional study and that patients with SIVD in this study had a mild degree of disease. In future studies, patients with more severe cognitive impairment should be included, and longitudinal observations should be conducted to explain the mechanism of disease development better.

In conclusion, our analysis showed that the fusiform and parahippocampal gyruses exhibited different subpatterns of asymmetry and lateralization in SVCI patients compared to healthy subjects and pre-SVCI patients. More interestingly, subjects' cognitive decline was correlated with the high pathological degree of asymmetry in specific areas of the fusiform and parahippocampal gyruses, indicating the possibility of using gray matter asymmetry as a biomarker for cognitive impairment in patients with SIVD.

## Data Availability Statement

The raw data supporting the conclusions of this article will be made available by the authors, without undue reservation.

## Ethics Statement

The studies involving human participants were reviewed and approved by the Ethics Committee of Chongqing Medical University. The patients/participants provided their written informed consent to participate in this study.

## Author Contributions

All authors have contributed to the manuscript and approved the final submission. RC: study design, data acquisition, analysis, interpretation, manuscript writing, and revision. LC: study design and statistical analysis. XL: data acquisition and revision. TL: study design and the approval of the final version for publication. JG and PJ: data acquisition.

## Conflict of Interest

The authors declare that the research was conducted in the absence of any commercial or financial relationships that could be construed as a potential conflict of interest.

## References

[1] Skrobot OA O'Brien J Black S Chen C DeCarli C Erkinjuntti T The vascular impairment of cognition classification consensus study. Alzheimers Dement. (2017) 13:624–33. 10.1016/j.jalz.2016.10.007 27960092

[2] Sachdev P Kalaria R O'Brien J Skoog I Alladi S Black SE. Diagnostic criteria for vascular cognitive disorders: a VASCOG statement. Alzheimer Dis Assoc Disord. (2014) 28:206–18. 10.1097/WAD.0000000000000034 24632990 PMC4139434

[3] Yu Y Liang X Yu H Zhao W Lu Y Huang Y. How does white matter microstructure differ between the vascular and amnestic mild cognitive impairment. Oncotarget. (2017) 8:42–50. 10.18632/oncotarget.13960 27992372 PMC5352131

[4] Fu Z Caprihan A Chen J Du Y Adair JC Sui J. Altered static and dynamic functional network connectivity in Alzheimer's disease and subcortical ischemic vascular disease: shared and specific brain connectivity abnormalities. Hum Brain Mapp. (2019) 40:3203–21. 10.1002/hbm.24591 30950567 PMC6865624

[5] Erkinjuntti T Subcortical ischemic vascular disease and dementia. Int Psychogeriatr. (2003) 15:23–6. 10.1017/S1041610203008925 16191213

[6] Sun YW Qin LD Zhou Y Xu Q Qian LJ. Abnormal functional connectivity in patients with vascular cognitive impairment, no dementia: a resting-state functional magnetic resonance imaging study. Behav. Brain Res. (2011) 223:388–94. 10.1016/j.bbr.2011.05.006 21605598

[7] Chen Z Liang X Zhang C Wang J Chen G Zhang H. Correlation of thyroid dysfunction and cognitive impairments induced by subcortical ischemic vascular disease. Brain Behav. (2016) 6:e00452. 10.1002/brb3.452 27127724 PMC4840667

[8] Kim JH Lee JW Kim GH Roh JH Kim MJ Seo SW. Cortical asymmetries in normal, mild cognitive impairment, Alzheimer's disease. Neurobiol. Aging. (2012) 33:1959–66. 10.1016/j.neurobiolaging.2011.06.026 21907459

[9] Habes M Erus G Toledo JB Zhang T Bryan N Launer LJ. White matter hyperintensities and imaging patterns of brain ageing in the general population. Brain. (2016) 139:1164–79. 10.1093/brain/aww008 26912649 PMC5006227

[10] Sarica A Vasta R Novellino F Vaccaro MG Cerasa A Quattrone A. MRI asymmetry index of hippocampal subfields increases through the continuum from the mild cognitive impairment to the Alzheimer's disease. Front Neurosci. (2018) 12:576. 10.3389/fnins.2018.00576 30186103 PMC6111896

[11] Zhu F Liu F Guo W Chen J Su Q Zhang Z. Disrupted asymmetry of inter- and intra-hemispheric functional connectivity in patients with drug-naive, first-episode schizophrenia and their unaffected siblings. EBioMedicine. (2018) 36:429–35. 10.1016/j.ebiom.2018.09.012 30241918 PMC6197719

[12] Derflinger S Sorg C Gaser C Myers N Arsic M Kurz. Grey-matter atrophy in Alzheimer's disease is asymmetric but not lateralized. J Alzheimers Dis. (2011) 25:347–57. 10.3233/JAD-2011-110041 21422522

[13] Low A Ng KP Chander RJ Wong B Kandiah N. Association of asymmetrical white matter hyperintensities and apolipoprotein E4 on cognitive impairment. J. Alzheimers Dis. (2019) 70:953–64. 10.3233/JAD-190159 31306121

[14] Cai S Chong T Zhang Y Li J von Deneen KM. Altered functional connectivity of fusiform gyrus in subjects with amnestic mild cognitive impairment: a resting-state fMRI study. Front Hum Neurosci. (2015) 9:471. 10.3389/fnhum.2015.00471 26379534 PMC4550786

[15] Catricalà E Della Rosa PA Parisi L Zippo AG Borsa VM Iadanza A. Functional correlates of preserved naming performance in amnestic Mild Cognitive Impairment. Neuropsychologia. (2015) 76:136–52. 10.1016/j.neuropsychologia.2015.01.009 25578430

[16] Yang H Wang C Zhang Y Xia L Feng Z Li D. Disrupted causal connectivity anchored in the posterior cingulate cortex in amnestic mild cognitive impairment. Front Neurol. (2017) 8:10. 10.3389/fneur.2017.00010 28167926 PMC5256067

[17] Pineault J Jolicoeur P Grimault S Bermudez P Brambati SM Lacombe J. Functional changes in the cortical semantic network in amnestic mild cognitive impairment. Neuropsychology. (2018) 32:417–35. 10.1037/neu0000466 29809032

[18] Wang Z Jia X Chen H Feng T Wang H. Abnormal spontaneous brain activity in early Parkinson's disease with mild cognitive impairment: a resting-state fMRI study. Front Physiol. (2018) 9:1093. 10.3389/fphys.2018.01093 30154730 PMC6102476

[19] Murayama N Ota K Kasanuki K Kondo D Fujishiro H Fukase Y. Cognitive dysfunction in patients with very mild Alzheimer's disease and amnestic mild cognitive impairment showing hemispheric asymmetries of hypometabolism on 8F-FDG PET. Int J Geriatr Psychiatry. (2016) 31:41–48. 10.1002/gps.4287 25820930

[20] Plessen KJ Hugdahl K Bansal R Hao X Peterson BS. Sex, age, and cognitive correlates of asymmetries in thickness of the cortical mantle across the life span. J. Neurosci. (2014) 34:6294–302. 10.1523/JNEUROSCI.3692-13.2014 24790200 PMC4004815

[21] Long XJ Jiang CX Zhang LJ. Morphological biomarker differentiating MCI converters from nonconverters: longitudinal evidence based on hemispheric asymmetry. Behav Neurol. (2018) 2018:3954101. 10.1155/2018/3954101 29755611 PMC5884406

[22] Kurth F Gaser C Luders E. A 12-step user guide for analyzing voxel-wise gray matter asymmetries in statistical parametric mapping (SPM). Nat Protoc. (2015) 10:293–304. 10.1038/nprot.2015.014 25591011

[23] Kurth F MacKenzie-Graham A Toga AW Luders E. Shifting brain asymmetry: the link between meditation and structural lateralization. Soc Cogn Affect Neurosci. (2015) 10:55–61. 10.1093/scan/nsu029 24643652 PMC4994843

[24] Núñez C Paipa N Senior C Coromina M Siddi S Ochoa S. Global brain asymmetry is increased in schizophrenia and related to avolition. Acta Psychiatr Scand. (2017) 135:448–59. 10.1111/acps.12723 28332705 PMC5407086

[25] Kurth F Thompson PM Luders E. Investigating the differential contributions of sex and brain size to gray matter asymmetry. Cortex. (2018) 99:235–42. 10.1016/j.cortex.2017.11.017 29287244 PMC5816677

[26] Zhu J Wang Y Wang H Cheng W Li Z Qian Y. Abnormal gray matter asymmetry in alcohol dependence. Neuroreport. (2018) 29:753–59. 10.1097/WNR.0000000000001027 29629981

[27] Román GC Erkinjuntti T Wallin A Pantoni L Chui HC. Subcortical ischaemic vascular dementia. Lancet Neurol. (2002) 1:426–36. 10.1016/s1474-4422(02)00190-4 12849365

[28] Galluzzi S Sheu CF Zanetti O Frisoni GB. Distinctive clinical features of mild cognitive impairment with subcortical cerebrovascular disease. Dement Geriatr Cogn Disord. (2005) 19:196–203. 10.1159/000083499 15677867

[29] Schmidt P Gaser C Arsic M Buck D Förschler A Berthele A. An automated tool for detection of FLAIR-hyperintense white-matter lesions in Multiple Sclerosis. Neuroimage. (2012) 59:3774–83. 10.1016/j.neuroimage.2011.11.032 22119648

[30] Guo Z Liu X Hou H Wei F Chen X Shen Y. (1)H-MRS asymmetry changes in the anterior and posterior cingulate gyrus in patients with mild cognitive impairment and mild Alzheimer's disease. Compr Psychiatry. (2016) 69:179–85. 10.1016/j.comppsych.2016.06.001 27423359

[31] Liao ZL Tan YF Qiu YJ Zhu JP Chen Y Lin SS. Interhemispheric functional connectivity for Alzheimer's disease and amnestic mild cognitive impairment based on the triple network model. J Zhejiang Univ Sci B. (2018) 19:924–34. 10.1631/jzus.B1800381 30507076 PMC6305256

[32] Ardekani BA Hadid SA Blessing E Bachman AH. Sexual dimorphism and hemispheric asymmetry of hippocampal volumetric integrity in normal aging and Alzheimer disease. AJNR Am J Neuroradiol. (2019) 40:276–82. 10.3174/ajnr.A5943 30655257 PMC7028613

[33] Kanwisher N Yovel G. The fusiform face area: a cortical region specialized for the perception of faces. Philos Trans R Soc Lond B Biol Sci. (2006) 361:2109–28. 10.1098/rstb.2006.1934 17118927 PMC1857737

[34] Zhao K Liu M Gu J Mo F Fu X Hong Liu C. The preponderant role of fusiform face area for the facial expression confusion effect: an MEG study. Neuroscience. (2020) 433:42–52. 10.1016/j.neuroscience.2020.03.001 32169552

[35] Kawagoe T Matsushita M Hashimoto M Ikeda M Sekiyama K. Face-specific memory deficits and changes in eye scanning patterns among patients with amnestic mild cognitive impairment. Sci Rep. (2017) 7:14344. 10.1038/s41598-017-14585-5 29085022 PMC5662773

[36] Pravatà E Tavernier J Parker R Vavro H Mintzer JE Spampinato MV. The neural correlates of anomia in the conversion from mild cognitive impairment to Alzheimer's disease. Neuroradiology. (2016) 58:59–67. 10.1007/s00234-015-1596-3 26400852

[37] Yang C Sun X Tao W Li X Zhang J Jia J. Multistage grading of amnestic mild cognitive impairment: the associated brain gray matter volume and cognitive behavior characterization. Front Aging Neurosci. (2016) 8:332. 10.3389/fnagi.2016.00332 28119601 PMC5222841

[38] Kang DW Lim HK Joo SH Lee NR Lee CU. Differential associations between volumes of atrophic cortical brain regions and memory performances in early and late mild cognitive impairment. Front Aging Neurosci. (2019) 11:245. 10.3389/fnagi.2019.00245 31551759 PMC6738351

[39] Sun P Lou W Liu J Shi L Li K Wang D. Mapping the patterns of cortical thickness in single- and multiple-domain amnestic mild cognitive impairment patients: a pilot study. Aging. (2019) 11:10000–10015. 10.18632/aging.102362 31756169 PMC6914405

[40] Owen AM Milner B Petrides M Evans AC. A specific role for the right parahippocampal gyrus in the retrieval of object-location: a positron emission tomography study. J Cogn Neurosci. (1996) 8:588–602. 10.1162/jocn.1996.8.6.588 23961986

[41] Aminoff E Gronau N Bar M. The parahippocampal cortex mediates spatial and nonspatial associations. Cereb Cortex. (2007) 17:1493–503. 10.1093/cercor/bhl078 16990438

[42] Luck D Danion JM Marrer C Pham BT Gounot D Foucher J. The right parahippocampal gyrus contributes to the formation and maintenance of bound information in working memory. Brain Cogn. (2010) 72:255–63. 10.1016/j.bandc.2009.09.009 19837500

[43] Mullally SL Maguire EA. A new role for the parahippocampal cortex in representing space. J Neurosci. (2011) 31:7441–9. 10.1523/JNEUROSCI.0267-11.2011 21593327 PMC3101571

[44] Bohbot VD Allen JJ Dagher A Dumoulin SO Evans AC Petrides M. Role of the parahippocampal cortex in memory for the configuration but not the identity of objects: converging evidence from patients with selective thermal lesions and fMRI. Front Hum Neurosci. (2015) 9:431. 10.3389/fnhum.2015.00431 26283949 PMC4522562

[45] Guo Y Zhang Z Zhou B Wang P Yao H Yuan M Grey-matter volume as a potential feature for the classification of Alzheimer's disease and mild cognitive impairment: an exploratory study. Neurosci Bull. (2014) 30:477–89. 10.1007/s12264-013-1432-x 24760581 PMC5562611

[46] Mitolo M Stanzani-Maserati M Capellari S Testa C Rucci P Poda R Predicting conversion from mild cognitive impairment to Alzheimer's disease using brain 1H-MRS and volumetric changes: a two- year retrospective follow-up study. Neuroimage Clin. (2019) 23:101843 10.1016/j.nicl.2019.101843 31071594 PMC6506639

